# Making the Next Generation of Therapeutics: mRNA Meets Synthetic Biology

**DOI:** 10.1021/acssynbio.3c00253

**Published:** 2023-09-06

**Authors:** Ahmet Hınçer, Recep Erdem Ahan, Ebru Aras, Urartu Özgür Şafak Şeker

**Affiliations:** † UNAM − Institute of Materials Science and Nanotechnology, National Nanotechnology Research Center, Bilkent University, Ankara 06800, Turkey

**Keywords:** mRNA-based therapeutics, mRNA vaccines, synthetic biology, self-assembled nanoparticles, logic gates

## Abstract

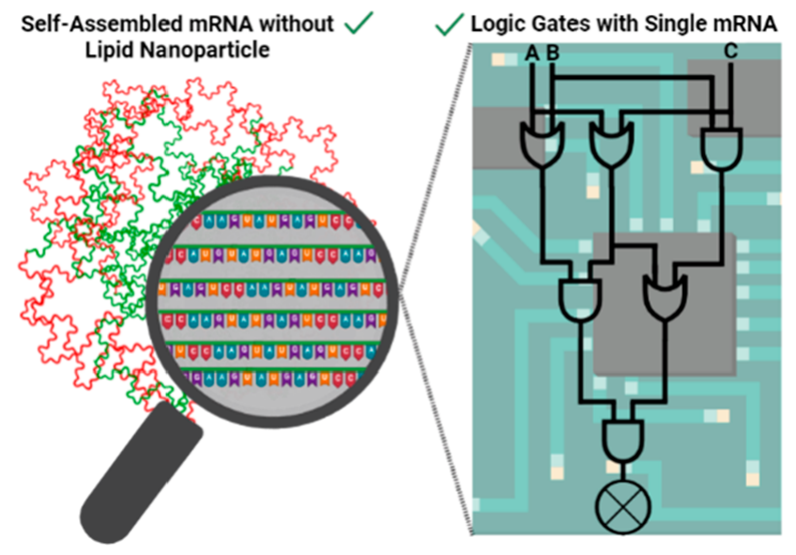

The development of mRNA-based therapeutics centers around the natural functioning of mRNA molecules to provide the genetic information required for protein translation. To improve the efficacy of these therapeutics and minimize side effects, researchers can focus on the features of mRNA itself or the properties of the delivery agent to achieve the desired response. The tools considered for mRNA manipulation can be improved in terms of targetability, tunability, and translatability to medicine. While ongoing studies are dedicated to improving conventional approaches, innovative approaches can also be considered to unleash the full potential of mRNA-based therapeutics. Here, we discuss the opportunities that emerged from introducing synthetic biology to mRNA therapeutics. It includes a discussion of modular self-assembled mRNA nanoparticles, logic gates on a single mRNA molecule, and other possibilities.

## Introduction

mRNA plays a pivotal role as the keystone of the central dogma, which is essential to the existence of life itself. Within the DNA-based genetic material of living systems, codes are concealed and utilized to generate a higher-level organization, facilitating the vitality of all organisms ranging from simple to complex. This organization revolves around proteins and their interactions with various organic and inorganic molecules, forming a multifaceted web of interactions that can be considered the wellspring of life. In this multiway interaction, the crucial message carrier is mRNA. As the carrier of this message, mRNA has garnered significant attention, leading to extensive studies of its manipulation and diverse applications.

A classic, distinguishing from newly explored noncanonical spliced counterparts (circular RNAs), mRNA molecule consists of four main components: the 5′ Cap, Untranslated Regions (UTRs) at both the 5′ and 3′ ends, the Coding Region, and Poly(A) Tails.^1−5^ The 5′ cap, an evolutionarily conserved modification in eukaryotic mRNA, assumes a role in initiating protein synthesis and acts as a protective barrier against exonuclease cleavage, which could otherwise result in early degradation.^6^ UTRs are noncoding regions flanking the coding sequence of mRNA that do not directly contribute to the protein sequence. The 5′ UTR serves as the entry point for ribosomes during translation, while the 3′ UTR contains regulatory elements influencing mRNA localization, protein conformation, and stability, thus playing significant roles in translation efficiency.^7−9^ The coding region is the essential segment responsible for protein translation, with codon usage affecting translation efficiency.^10^ The utilization of frequently occurring synonymous codons and codon usage bias has been found to enhance translation magnitude by reusing the same tRNA molecule or increasing the abundance of tRNA molecules in the vicinity of ribosomes.^11−13^ Finally, the poly(A) tail, consisting of repeated adenosine nucleotides, plays a crucial role in mRNA translation and stability by interacting with the 5′ cap and facilitating circularization.^14^

To unleash the potential of mRNA as a therapeutic agent, extensive research has focused on understanding, improving, and optimizing the main components of classic mRNA molecules. Various capping methodologies have been developed, including antireverse cap analogs (ARCAs) and CleanCap.^15−18^ In many applications, human α- and β-globin gene UTRs have been widely utilized.^10,19^ UTRs have also been engineered based on secondary structures to provide stability or preferential translatability, such as riboswitches, for therapeutic purposes.^7,9,20^ The impact of poly(A) tail length on expression and stability has been extensively studied, determining the optimum length to minimize mRNA decay caused by tail shortening and hyperadenylation.^21,22^ In addition to mRNA component studies, modifications of nucleosides, such as pseudouridine and N1-methylcytosine, have been established to create more stable mRNAs thanks to reduced activation of protein kinase R (PKR) which can decrease the translation capacity of cells via PKR-mediated phosphorylation of a subunit of translation initiation factor, low activation of RNase L and increased resistance to this RNase. Additionally, these modifications can diminish the ability of mRNAs to activate proinflammatory cytokine release, induction of costimulatory molecules by interacting with most of the TLRs (especially TLR3, TLR7, and TLR8).^23−25^ However, effective mRNA manipulation alone is insufficient to achieve the desired efficacy and eliminate potential side effects without proper delivery methods. Although naked mRNA delivery is possible, it is primarily impractical due to degradation by nucleases, preventing a significant portion of mRNA from reaching the target cells. Moreover, most mRNAs that do reach enter the cells via caveolae, which is a type of lipid raft, and these mRNAs tend to accumulate in lysosomes, which leads to degradation. Consequently, only a minimal amount of mRNA escapes the lysosomes and reaches the translation machinery.^26−30^ To address these challenges and establish efficient and safe mRNA delivery to target cells, various methods have been developed, including lipid nanoparticles (LNPs), polymers, and cell-penetrating peptides (CPPs).^31−35^ Lipid nanoparticles, one of the most advanced and extensively studied mRNA delivery systems, facilitate cell transfection through endocytosis. They are composed of ionizable cationic lipids, phospholipids, cholesterol, and polyethylene glycol (PEG). Among polymers, polyethylenimine (PEI) and poly(β-amino) esters (PBAE) are the most commonly used materials. Protamines, a type of cationic peptide that is used as CPP, have been extensively investigated as promising tools for intracellular mRNA delivery. Protamines effectively protect the mRNA cargo via binding from degradation by ribonucleases and facilitate its safe transport into the cytoplasm, making them commonly employed in gene therapy and mRNA-based therapeutics.^26,34,36−40^

Advancements in mRNA molecules and delivery techniques have unlocked the potential to translate mRNA technology from laboratories to medicine. Numerous designed mRNA therapeutics have entered different stages of clinical trials, offering solutions for a wide range of health issues. Phase 3 trials for three vaccine candidates against COVID-19 have been completed, while vaccine clinical trials for other viruses, such as rabies, influenza, and acute HIV infection, are still ongoing. Clinical trials of different mRNA-based strategies are also underway for cancer immunotherapies targeting cancers like melanoma, glioma, and Hodgkin lymphoma; genetic disorders such as cystic fibrosis; metabolic disorders exemplified by type 2 diabetes; and cardiovascular diseases. These medications are currently in phase 1 and 2 trials, showcasing the promising potential of mRNA therapeutics.^41^

Here, we have discussed that the convergence of mRNA, the message carrier of life, and synthetic biology, the redesigning approaches of life, opens up possibilities and applications. The meeting of mRNA and synthetic biology, coupled with hypothetical *in silico* advancements, holds immense potential for emerging applications and strategies in mRNA therapeutics over the next two decades.

## Current State of mRNA Therapeutics

The field of mRNA therapeutics has made significant strides in terms of the fundamental role of mRNA in the central dogma, which is that the encoded protein is expressed. Several applications of mRNA therapeutics revolve around this key step, translation. These applications include protein replacement therapies for chronic diseases such as diabetes, anemia, and myocardial infarction, where mRNA delivery enables the expression of specific proteins at the target site. mRNA-based cancer immunotherapies utilize mRNA to express cancer-specific antigens, triggering an immune response against malignant tumors or transfecting immune cells with mRNA encoding chimeric antigen receptors. Additionally, mRNA is used for genome engineering by expressing nucleases such as ZFN, TALEN, or Cas9 to induce targeted modifications, as well as for genetic reprogramming of cells to enhance pluripotency.^10,36,38,42−45^ On the other hand, antisense oligonucleotide (ASO) and RNA interference (RNAi) therapies are distinct from mRNA-based therapeutics. Instead of directly delivering mRNA to encode specific proteins, ASO and RNAi therapies work by modulating the levels of existing mRNA molecules within cells. In ASO therapy, short single-stranded nucleotides are designed to bind to complementary RNA sequences, forming a DNA-RNA duplex. This duplex is recognized by the enzyme RNase H, which cleaves the RNA strand, resulting in the degradation of the target mRNA. This down-regulates the expression of the targeted gene. In RNAi therapy, single- or double-stranded RNA molecules are introduced into the cell. These RNA molecules are then processed by the RNA-induced silencing complex (RISC) and guide the RISC to target specific mRNA molecules, leading to their degradation and subsequent downregulation of the corresponding gene expression. Both ASO and RNAi therapies are valuable tools for gene regulation and have shown potential for various therapeutic applications. ASO therapy, in particular, can be used for exon skipping to correct mutations that occur on a specific exon, helping to restore the open reading frame and potentially treat genetic disorders. These approaches offer alternative ways to modulate gene expression and hold promise for treating a wide range of diseases.^46^

The development of mRNA-based COVID-19 vaccines, which involve the expression of viral antigens in the body through *in vitro* transcribed mRNA, is another prominent example rooted in this principle. The progress made in studies like these has provided significant momentum toward the commercialization of mRNA-based biologics. However, there are still technological challenges that need to be addressed before the full potential of these applications can be realized.

## The Safety of mRNA-Based therapeutics

Using mRNA as a therapeutic agent is indeed not a new concept, as demonstrated by Robert W. Malone in 1989 when he showed that lipid-mixed mRNA can be translated into cultured eukaryotic cells. However, despite this groundbreaking research, the progress and utilization of mRNA-based therapeutics have taken nearly three decades. The primary reasons behind this delayed progress were the limited understanding of mRNA properties.^47^

mRNA is inherently susceptible to degradation by ubiquitous RNases and hydrolyzation at pH values higher than 6. Additionally, without an appropriate carrier, the chances of mRNA passing through an anionic cell membrane to express encoded proteins in the cell’s cytoplasm were quite low, with less than a 1/10000 molecules probability.^41^ Moreover, the immunogenic nature of mRNA posed challenges in conducting *in vivo* studies effectively.^48^

Due to these unique characteristics, extensive research focused on developing delivery agents and implementing good manufacturing practices for producing *in vitro* transcribed mRNA as a therapeutic. Addressing the challenges associated with mRNA stability, cellular uptake, and immunogenicity has become critical for advancing the field of mRNA-based therapeutics.

The use of Good Manufacturing Practice (GMP) for mRNA, as required by the EMA and FDA is crucial for ensuring the safety and quality of therapeutic products.^49,50^ GMP guidelines necessitate animal-component-free reagents or, if necessary, extensive analysis and testing to prevent accidental safety issues during traditional therapeutic production.

In the case of *in vitro* transcription of mRNA, a DNA-based template, such as plasmid DNA or PCR amplicons, is utilized. The production process involves several steps according to the scale of the study, including DNase treatment, LiCl precipitation, and reverse phase FPLC, to purify single-stranded mRNAs and remove any residual DNA, enzymes, NTPs, and double-stranded mRNAs. These stringent purification steps are essential to ensure that the final mRNA product is contaminant-free.^36^

Compared to other platforms such as live viruses, viral vectors, inactivated viruses, and protein subunits, the mRNA production process is relatively short, which reduces the biological and chemical contamination risk for mRNA-based therapeutics. For instance, the mRNA vaccines developed by BionTech for SARS-CoV-2 produced in institutes where all GMP standards are met.^51^ The required standard testing for contaminants and toxic elements is applied for every batch produced and reported accordingly. In this quality control testing, the final product is approved as DNA-free.

On the other hand, the development of these vaccines involves a strategic design to ensure that their lipid carriers exhibit a preference for specific tissues. This selectivity is achieved through tropism assessments, which are facilitated by studying lipid libraries.^33^ As a result, when mRNA-laden lipid nanoparticles (LNPs) are administered, their biodistribution shows that approximately 75% of them remain at the injection site, around 21% accumulate in the liver, and less than 1% are found in the spleen.

It is important to note that the nature of mRNA molecules inherently leads to their elimination after antigen presentation in the body. This process is primarily facilitated by cellular RNases, as well as their relatively short half-life. Unlike DNA vaccines, these mRNA molecules do not enter the nucleus for translation, meaning they are not integrated into the human genome.^41^ Therefore, there is no risk of long-term genetic alteration through the administration of mRNA-based vaccines.

Comprehensively, the carefully engineered lipid carriers in these vaccines, coupled with the properties of mRNA molecules, provide a safe and effective means of triggering immune responses without introducing any permanent genetic changes to the human body.

Clinical data from mRNA vaccines for SARS-CoV-2 have shown that the adverse effects are generally mild and of short duration, indicating that mRNA therapeutic approaches are notably safe.^52^

Overall, the stringent manufacturing processes and the clinical safety data support the safety of mRNA-based therapeutics, making them a promising and secure approach for various medical applications including vaccines and gene therapies. Despite the pseudoscience saga, mRNA-based therapeutics do not have a potential to get fused into the cellular genome due to the absence of reverse transcriptase enzymes in human cells. All of the—unfortunately published—experiments to support this pseudoscience saga are misleading or poorly designed in a manipulative manner, which have eventually failed. Science aims to investigate the facts with properly designed experimental setups. Designing experiments missing control groups or poor/manipulative designs to support a “believed” hypothesis is not a definition of science.

## Advancing mRNA Technology: Current Gaps and Studies

The current clinically approved mRNA technology lacks specificity in targeting specific cell types, tunability to adjust product expression, and long-lasting profiles within cells. As a result, a large total amount of injected mRNA and frequent injections are often required. Researchers are actively investigating approaches to address these limitations and advance the field of mRNA therapeutics.

One area of research focuses on the specific delivery of mRNA therapeutics to particular cell types, with a particular focus on optimizing lipid nanoparticle (LNP) formulations. LNPs, composed of ionizable cationic lipids, phospholipids, cholesterol, and poly(ethylene glycol) (PEG), are crucial for the effectiveness of mRNA vaccines. However, they can cause hypersensitivity reactions and immune-mediated adverse effects due to individual variability. Optimization of LNPs is necessary to reduce accumulation in specific areas of the body and prevent activation of the complement system due to LNP components.^53,54^ While many researchers have adopted the approach of screening lipid libraries to find suitable lipid mixtures that desired cell types can take up, there are also promising “out-of-the-box” ideas, such as decorating LNP surfaces with proteins, that could help restrict the cell tropism of mRNA-laden LNPs.^33,55−59^ One approach involves immobilizing antibodies on LNPs using membrane-anchored scFv called ASSET, enabling the targeting of LNPs to different white blood cells expressing CD3, CD5, or beta 7 integrin.^57^ Another study demonstrated the targeting of T cells by decorating the surface of LNPs carrying mRNA encoding information for reprogramming them via CD5-specific antibodies, facilitating the *in vivo* creation of CAR-T cells.^60^

The regulation of mRNA therapeutics is further influenced by cis- and trans-acting elements in alternative splicing, trans-acting RNA binding proteins, and miRNA-dependent regulations of endogenous mRNA. These elements provide additional control points for the specificity of mRNA therapeutics. Alternative splicing plays a significant role in regulating gene expression. The location of the mRNA or differences in specific protein levels can directly impact the fate of the expressed protein.^61^ A classic example is Fibronectin (FN1), where alternative splicing mechanisms influence its solubility and cellular localization, affecting its function in the body.^62^

To achieve cell-type-specific expression, mRNA molecules can be engineered to carry specific regulatory elements. One application of alternative splicing is the splicing-linked expression design (SLED), which allows for a higher level of control over protein production. By utilizing cell-type-specific splicing sites, SLED creates frameshifts in the coding sequence, leading to the expression of different proteins based on the specific splicing properties of the cell type.^63^

Additionally, miRNAs play a crucial role in post-transcriptional gene regulation. Lockhart et al. harnessed the natural function of miRNAs to control p27 encoding mRNA degradation, specifically in endothelial cells. Through this approach, they demonstrated targeted expression in vascular smooth muscle cells.^64^

Overall, these intricate regulatory mechanisms provide precise control over the expression of mRNA therapeutics, enabling specific targeting of desired cell types and tissues. Such sophisticated approaches hold promise for developing highly tailored and effective treatments for various diseases. Moreover, protein-based mRNA delivery shuttles and eukaryotic toehold switches offer additional avenues to enhance the specificity of administered mRNA therapeutics through further research.^65,66^

The tunability of mRNA action inside the body after injection is a crucial aspect of ensuring the safety of mRNA-based therapeutics. This need for external control of complex biologics was highlighted by a fatal incident related to Her-2 specific adoptive cell therapy, emphasizing the importance of carefully managing potential serious side effects.^67^ To address this issue, researchers have explored approaches, such as engineering self-amplifying RNA molecules that can be regulated and titrated with FDA-approved small molecules like trimethoprim, enabling fine-tuning of mRNA expression levels and therapeutic effects.^68^

Enhancing the durability of administered mRNA inside transfected cells can reduce the amount of mRNA required, while still maintaining therapeutic efficacy. As mentioned earlier in this article, current research aims to improve the half-life of transfected mRNA by engineering different regions of the mRNA molecule, including the 5′ UTR, 3′ UTR, 5′ cap, and Poly(A) tail. In a notable study by Wesselhoeft et al., a novel approach was introduced involving the circularization of IRES-containing RNA to create circular RNA (circRNA). They found that circularized coding RNA with IRES exhibited production efficiency similar to that of linear mRNAs. In a follow-up study, circRNAs showed inertness toward innate immunity elements such as Rig-I and did not lead to overexpression of TLRs.^5^ Additionally, Chen et al. demonstrated that the rational design of circRNAs could increase their translation capacity and overall durability. These findings provide valuable insights into optimizing mRNA therapeutics and enhancing their potential for long-lasting and effective treatments.^1^

These ongoing research efforts hold promise for addressing the specificity, tunability, and durability of mRNA therapeutics, paving the way for safer and more effective treatments with lower side effects and reduced injection frequency.

## Introducing Synthetic Biology to mRNA Technology

Efforts to narrow the gaps in mRNA technology have indeed been centered on harnessing the natural function of mRNA molecules, especially their translation process within cells. Researchers aim to achieve their desired therapeutic outcomes by directly manipulating mRNA or by advancements in delivery systems.

In the realm of mRNA therapeutics, the conventional focus has been on leveraging the natural properties of mRNA for the last 20 years. However, by adopting a synthetic biology mindset, researchers can repurpose or redesign these natural properties by incorporating unnatural components. This combination of natural and synthetic elements has the potential to significantly enhance the capabilities of mRNA therapeutics significantly. While this transition may seem substantial, it is more accurately described as a combination of approaches. This blending of natural and synthetic approaches, which lies at the core of synthetic biology, holds promise as a new frontier for mRNA therapeutics in the coming decades. This work introduces two distinct strategies that illustrate how this combination can revolutionize mRNA-based applications. These strategies showcase how this approach can enhance the efficacy, specificity, and safety of mRNA therapeutics, making it the forerunner of novel treatment options for a wide range of diseases.

### Self-Assembled mRNA without Lipid Nanoparticles

An mRNA therapeutic consists of two main components: the mRNA itself and the delivery agent. These two parts have been extensively optimized to achieve the desired therapeutic outcomes in various applications. The mRNA compartment is responsible for encoding the desired protein, while the delivery agent compartment ensures the successful delivery of the mRNA to target cells without degradation by nucleases. Both compartments are critical in the development of mRNA therapeutics, and optimizing both components is necessary to achieve effective and efficient mRNA delivery for the desired therapeutic outcomes.

By adoption of a synthetic biology approach, it is possible to streamline the optimization process by using mRNA molecules as their own delivery agents. This approach reduces the number of optimization segments from two to one. Nucleic acids, such as DNA and RNA, have been extensively studied for their ability to bind and deliver other molecules, making them promising candidates for use as delivery agents.^70−73^ Previous research has demonstrated the ability of self-assembled mRNA molecules to undergo translation despite their complex structure, which can make them challenging for ribosomes to access.^74^ By combining the unnatural loadability and self-assembly properties of mRNA with its natural translatability feature, we may be able to create a single, optimized compartment for mRNA therapeutics. This could simplify the development and optimization process, potentially leading to more efficient and effective therapies for various medical conditions.

Furthermore, noncovalent interactions between nucleotides in a single mRNA molecule can provide a desired shape recognizable by specific target cell receptors, similar to aptamer-protein interactions.^75−83^ This precise targeting and delivery mechanism can improve the specificity and efficiency of mRNA-based therapies. The ability to control the spatial structure of nucleic acids through noncovalent interactions is an exciting area of research with promising applications in different medical fields. This feature ensures the targetability of a single-unit mRNA therapeutic.

**Figure 1:**
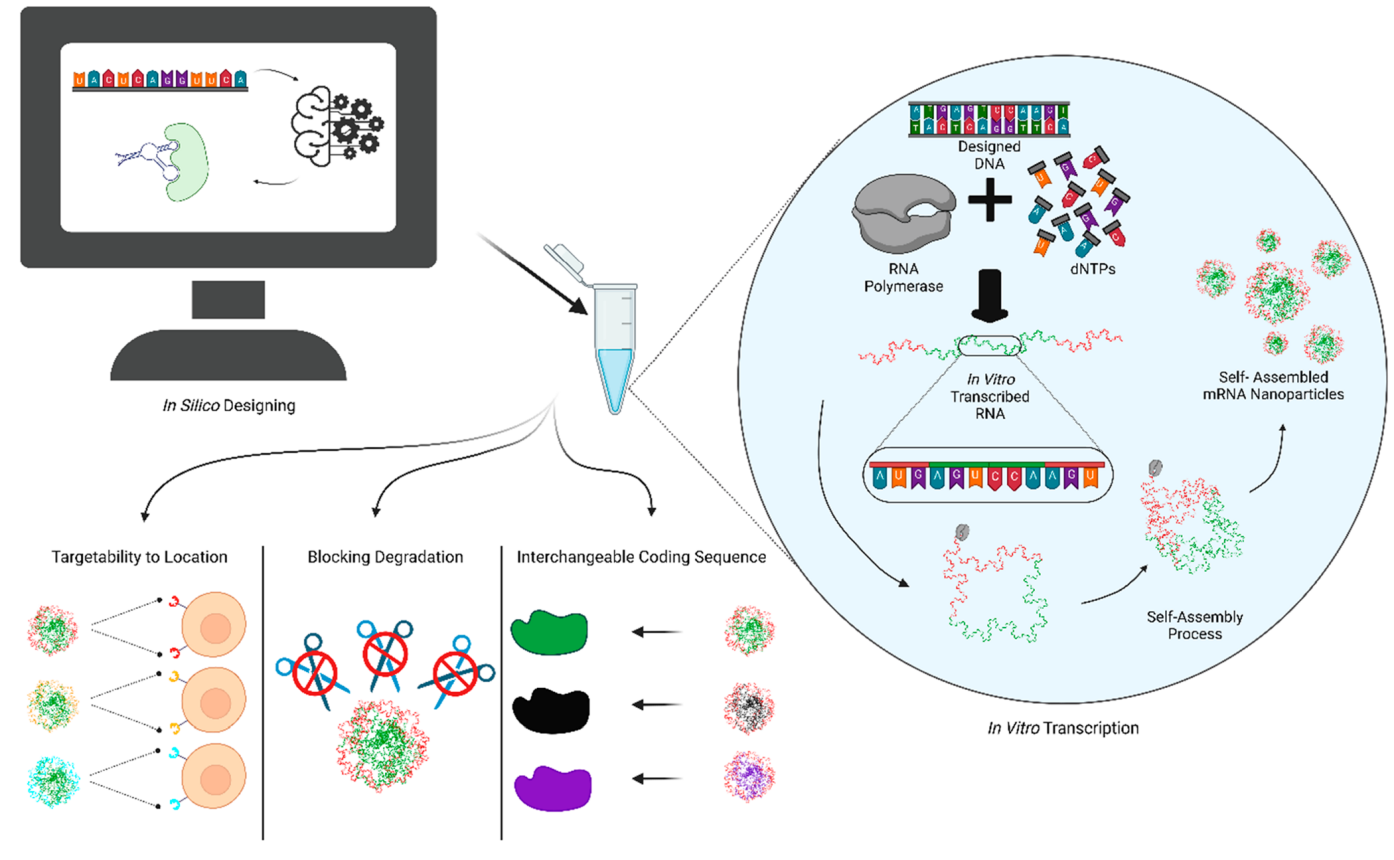
Design of self-assembled mRNA nanoparticles. *In silico* decoration of the mRNA sequence is crucial to reach aims for targetability, nondegradability, and interchangeability. While the designed mRNA is being transcribed *in vitro*, the self-assembly process occurs synchronously. Outside of these, self-assembled mRNAs would be changeable aptamers to trigger entry into specific cell types. The compactness of the particles would prevent degradation. Inside of the nanoparticles, the transitions would be switched according to the application of interest. The modularity of this design would provide acceleration for mRNA-based therapeutics. This figure is created with BioRender.com.^69^

To create an mRNA scaffold that contains regions that fold as aptamers to bind to receptors at the target site, assembles into nanoparticles to prevent mRNA degradation, and incorporates interchangeable coding sequences for various applications, further improvement of *in silico* methodologies for controlling the mRNA spatial structure is necessary (Figure 1). This integration of synthetic biology in mRNA therapeutics is crucial and promising, as it allows for the combination of natural parts to create an unnatural system, optimizing the delivery and translation of mRNA for enhanced therapeutic outcomes.

### Genetic Logic Gates with a Single mRNA Molecule

Another aspect of the synthetic biology approach involves redesigning natural systems by introducing synthetic compartments, allowing complete control over these systems. *In silico* engineering of non-native parts can be used to reconstruct basic natural systems, incorporating both natural and unnatural elements to create new systems with higher levels of control and usability.

Toehold switches, both prokaryotic and eukaryotic, serve as examples of such parts that rely on native mechanisms of the central dogma but with unconventional control machinery.^66,84,85^ These synthetic switches provide precise control over gene expression and have been studied for various applications. Biological circuits that create logical networks have also been explored, as they can be designed to respond to specific inputs and produce the desired outputs. Such systems have been investigated for diagnostic and therapeutic purposes, although most studies have focused on DNA-based methodologies.^86−88^ However, DNA-based circuits have certain drawbacks, including nontemporary activation, the need for nuclear localization upon cell entry, and the risk of genomic integration.^10,89−91^ Considering these limitations, innovations in nucleic acid architecture, particularly with advancements in CRISPR-Cas technology, the eToehold concept, and the discovery of trans-acting cap-independent translational elements, may allow for a shift in focus from DNA-based to RNA-based biological circuit-based diagnostic and therapeutic tools.^66,92−96^ Recently, the research conducted by the Santangelo and Gersbach laboratories has showcased a new approach to mRNA-based therapeutics. They demonstrated that mRNAs encoding chimeric dCas9 protein loaded to an LNP carrier could transcriptionally activate gene expression directly from the DNA-based material, genome. This activation is achieved through precise targeting with the help of sgRNA companions *in vivo*.^97^ This development opens up the possibility of using RNA-targeting chimeric dCas13 proteins to control translation and potential RNA-based circuit designs with the same synergy of trans-acting cap-independent translational elements, potentially in *in vivo* studies.

While there may be challenges associated with using RNA, such as stability and compatibility with the working principle of the designed circuit since the principle of it may include cleavage of RNA leading to the whole degradation of uncapped or de-adenylated RNA, incorporating a DNA-based circuit into a single mRNA molecule and mitigating the drawbacks associated with DNA could hold promise.^98,99^ This approach opens up possibilities for more sophisticated applications of RNA-based circuitry in diagnostic and therapeutic contexts.

#### AND Gate

**Figure 2:**
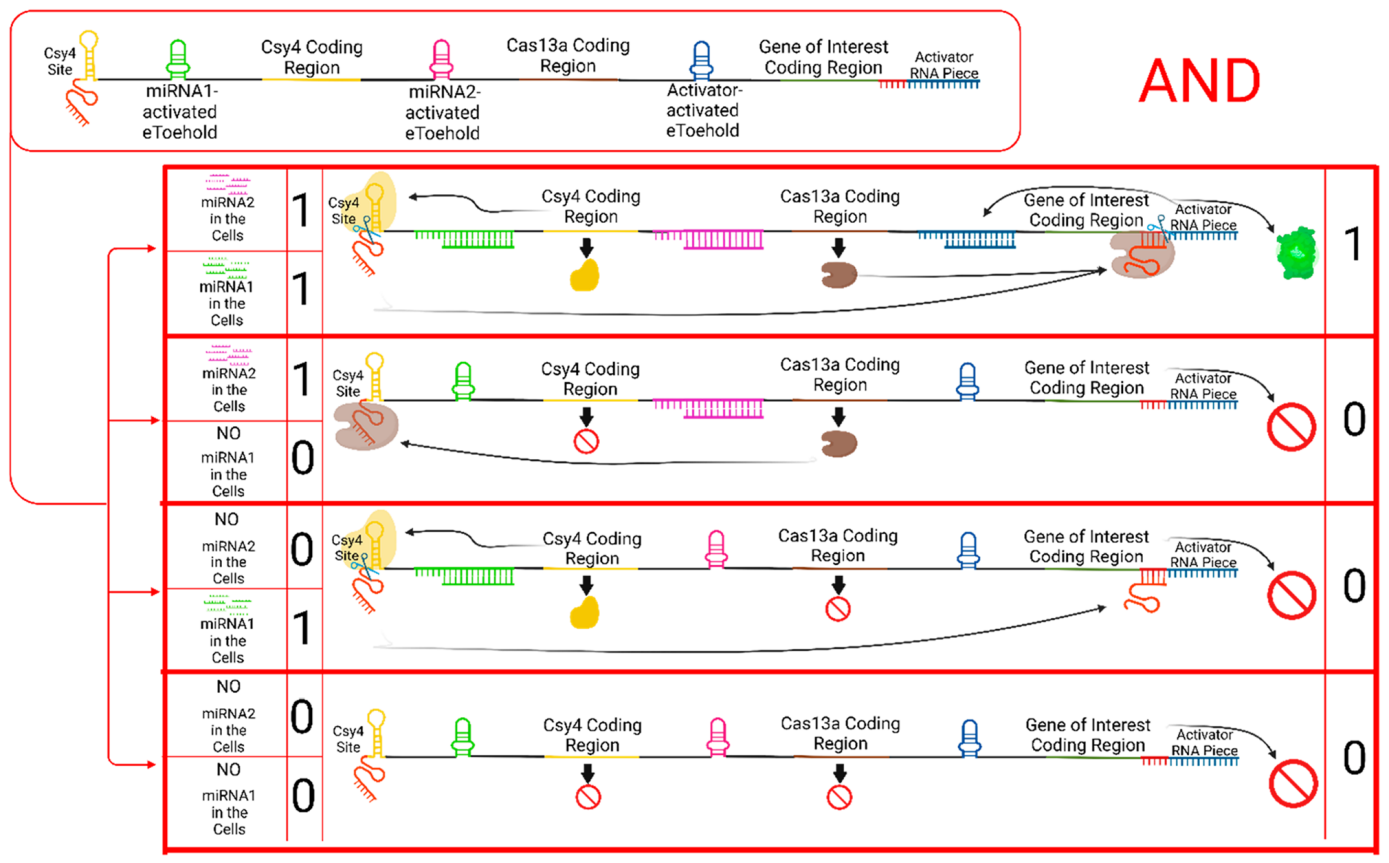
AND gate via a single mRNA molecule. After delivery of the mRNA molecule (top left) to the cells, the presence of miRNA1 in the cell leads to the translation of Csy4 enzyme to cut its recognition site, whereas the presence of miRNA2 results in the expression of Cas13a enzyme. The cut of Csy4 releases the crRNA for Cas13a, and it results in the disengagement of the activator RNA piece to activate the translation of the gene of interest. The only condition required to express the gene of interest is that both of the miRNAs must be found in the cell. This figure is created with BioRender.com.^69^

To design a single mRNA molecule AND gate circuit, three different eToehold regions are required to control the expression of the Csy4 nuclease, the Cas13a nuclease, and the gene of interest (Figure 2). This circuit utilizes two miRNA inputs, and their combinations are specific to the target cell.

The first miRNA, miRNA1, activates the expression of Csy4 CRISPR nuclease. The second miRNA, miRNA2, initiates translation of the Cas13a nuclease. In target cells, where both miRNAs are present, both enzymes are expressed. The Csy4 nuclease digests the mRNA from the Csy4 recognition site, leading to the release of the crRNA of Cas13a, which is represented by the red piece at the beginning of the mRNA. The Cas13a nuclease, together with its crRNA, can then cleave the recognition site of the crRNA, represented by the red region at the beginning of the activator RNA piece. Upon release, the activator RNA piece activates the expression of the gene of interest.

The key feature of this circuit is that if one of the miRNAs is missing in the cell, the system cannot produce the final product, which is the gene of interest. This design ensures that the expression of the gene of interest is tightly controlled and dependent on the presence of both miRNAs, making it specific to the target cell.

Overall, this single mRNA molecule AND gate circuit provides a mechanism to precisely regulate the expression of the gene of interest in a target cell based on the combination of specific miRNA inputs.

#### OR Gate

**Figure 3:**
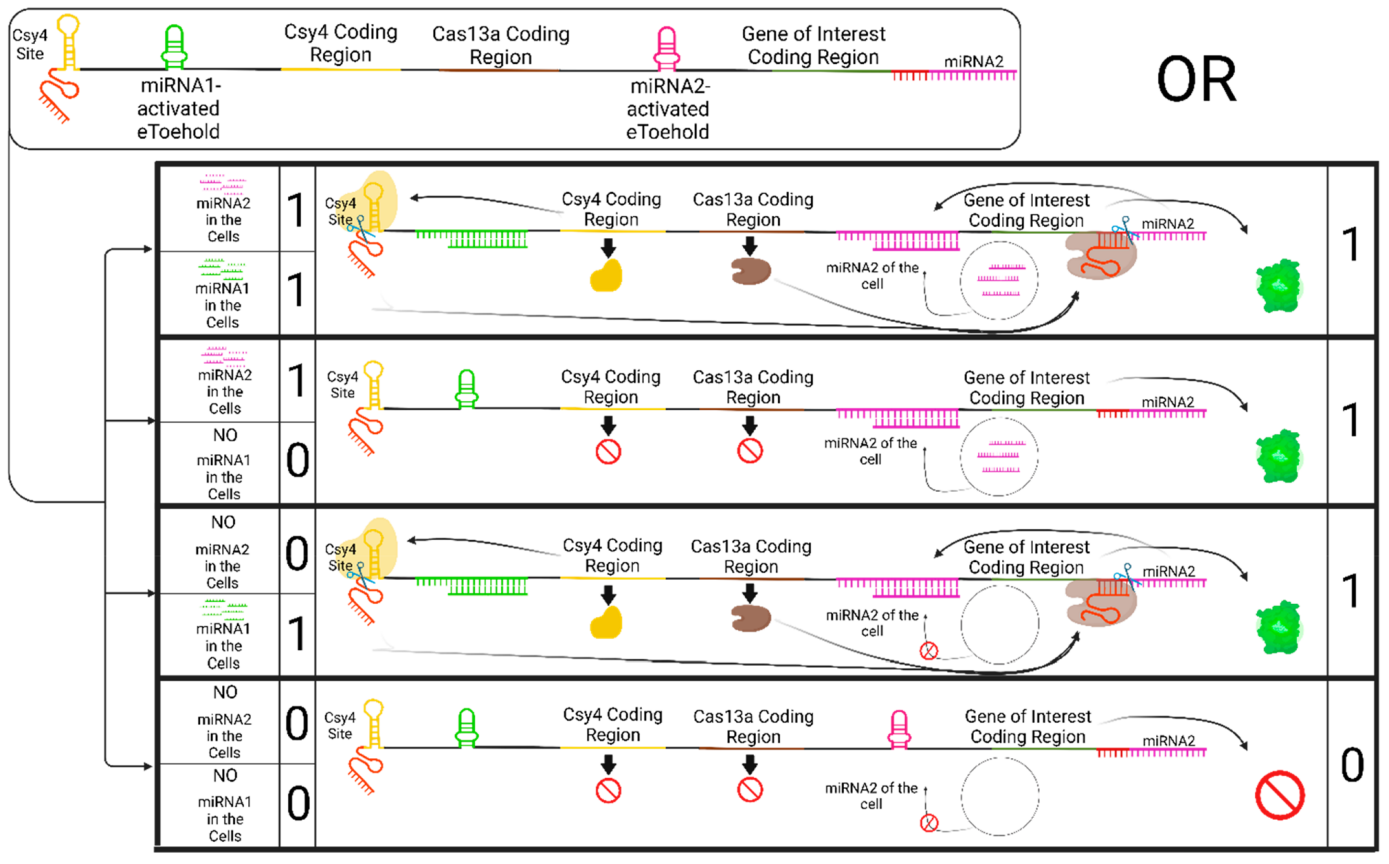
OR gate via a single mRNA molecule. After delivery of the mRNA molecule (top left) to the cells, the presence of miRNA1 in the cell leads to the translation of both Csy4 and Cas13a enzymes. The cut of Csy4 releases the crRNA for Cas13a, which results in disengagement of the miRNA2 piece to activate the translation of the gene of interest. The only condition not leading to the expression of the gene of interest is that both of the miRNAs must be absent in the cell. Expression of the gene of interest would occur with the presence of either miRNA1, miRNA2, or both in the cell. This figure is created with BioRender.com.^69^

To construct an OR gate using a single mRNA molecule, two different eToehold regions are required: One for controlling the expression of Csy4 and Cas13a simultaneously and the other for the production of the gene of interest (Figure 3). This circuit also utilizes two miRNA inputs, similar to the AND gate previously described, but in this scenario, the presence of either miRNA is sufficient to activate the expression of the gene of interest.

Specifically, miRNA1 activates the expression of both Csy4 and Cas13a nucleases simultaneously. On the other hand, miRNA2 initiates the translation of the gene of interest and is located at the end of the mRNA molecule (represented by the purple piece), in addition to those present in the cell itself, if it contains miRNA2. When miRNA1 is present, it triggers the expressions of Csy4 and Cas13a, leading to the cleavage of miRNA2 at the end of the mRNA via a similar mechanism, as shown in the AND gate circuit. This cleavage event allows the release of miRNA2, which can then start translation of the gene of interest. The translation of the gene of interest is controlled by the eToehold region activated by miRNA2.

In this OR gate circuit, the presence of either miRNA1 or miRNA2 is sufficient to activate the desired function, which is the expression of the gene of interest. However, if both miRNAs are absent in the cell, then this circuit remains inactive.

Basically, this single mRNA molecule OR gate circuit enables the activation of the gene of interest in response to the presence of either miRNA1 or miRNA2, providing flexibility and versatility in controlling gene expression based on the specific miRNA inputs.

#### XOR Gate

**Figure 4:**
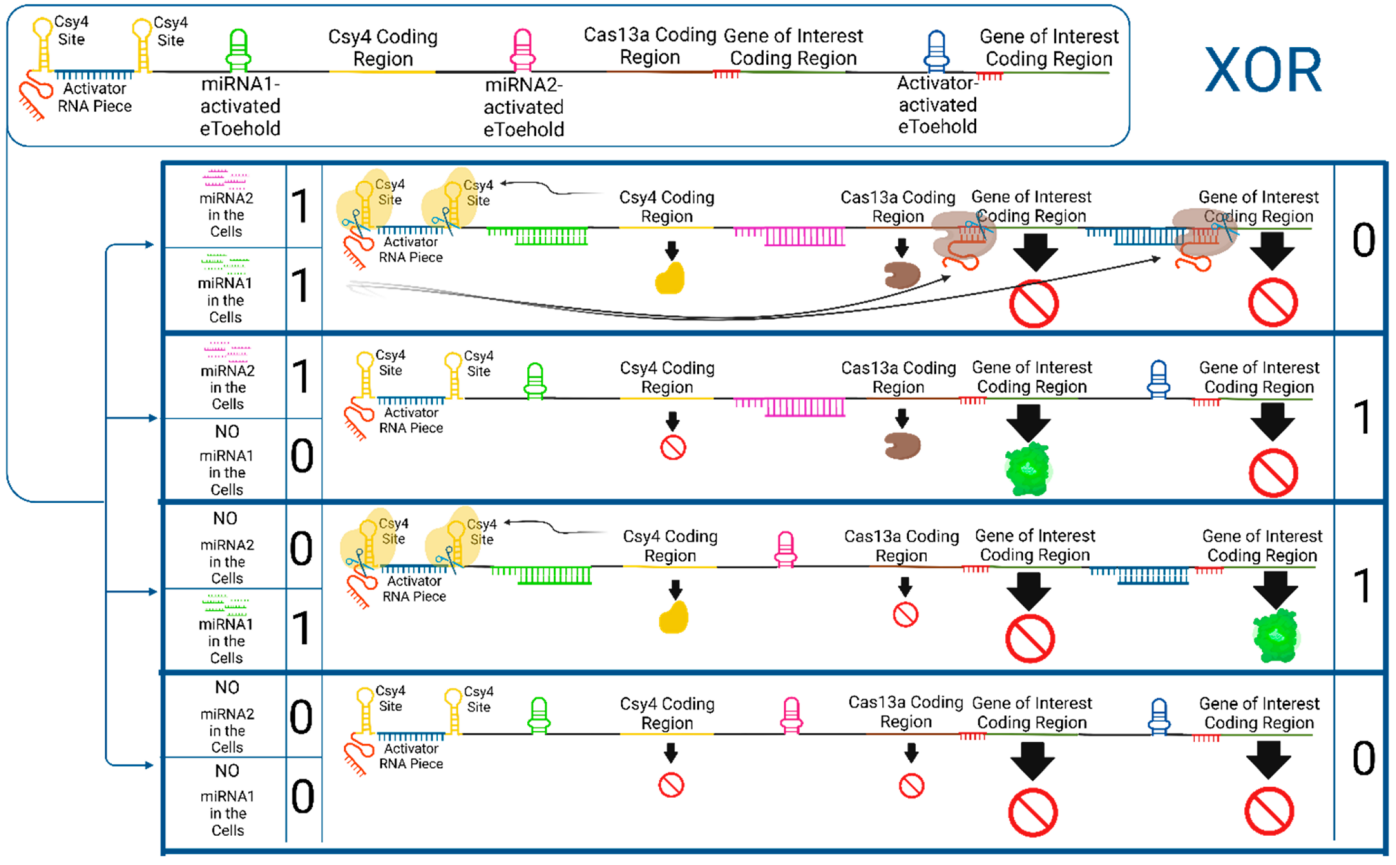
XOR gate via a single mRNA molecule. After delivery of the mRNA molecule (top left) to the cells, the presence of miRNA1 in the cell leads to the translation of Csy4 enzyme. The cut of Csy4 releases the crRNA (red piece at the beginning of mRNA) and activator RNA piece, which can lead to expression of the gene of interest, only there is no miRNA2 in the cell since miRNA2 starts the Cas13a expression. miRNA2 can also initiate the gene of interest production simultaneous with Cas13a, however this initiation requires no miRNA1 in the cells to release crRNA. The presence and absence of miRNAs at the same time results in no expression of the gene of interest. This figure is created with BioRender.com.^69^

To build an XOR gate using a single mRNA molecule, three distinct eToehold regions are necessary: one to regulate the expression of Csy4, another for simultaneous production of Cas13a and the gene of interest, and a third for the translation of the gene of interest alone (Figure 4). Additionally, this circuit involves two miRNA inputs, similar to the AND and OR gates discussed earlier. However, in this case, only one type of miRNA is required to activate the expression of the gene of interest. Simultaneous presence or absence of both miRNAs will deactivate the circuit.

When miRNA1 is present, it triggers expression of the Csy4 gene. The Csy4 nuclease can then cleave both Csy4 recognition sites, leading to the release of the activator RNA piece and crRNA. In the absence of miRNA2 in the cell, Cas13a remains inactive, and crRNA cannot target any of its recognition sites. The activator RNA molecule initiates the expression of the gene of interest. However, in the presence of miRNA2s, Cas13a combines with crRNA and cleaves both of its recognition sites. Consequently, the gene of interest is removed from the translation initiation sites, preventing its expression. Conversely, if only miRNA2s are present, then it directly initiates the production of both Cas13a and the gene of interest. However, it cannot cleave the recognition sites of the Cas13a and crRNA combination.

## Conclusion

In drawing to a close, the current development of mRNA-based therapeutics primarily revolves around harnessing the natural role of mRNA as a message carrier of life and optimizing its translation and delivery to target cells. While improving conventional mRNA therapies is crucial, it represents just a fraction of what is possible. Embracing a synthetic biology mindset and capitalizing on *in silico* advancements offer a vast and untapped potential to revolutionize mRNA-based applications. This shift in perspective opens the door to exploring uncharted territories and uncovering hidden opportunities in the realm of mRNA therapeutics, unveiling the immense possibilities that lie beyond the surface. With continued research and innovation, the future of mRNA-based therapeutics holds tremendous promise for advancing medical treatments and improving patients’ lives.
